# Engineered Chimera Protein Constructs to Facilitate the Production of Heterologous Transmembrane Proteins in *E. coli*

**DOI:** 10.3390/ijms25042354

**Published:** 2024-02-16

**Authors:** Adeyemi Ogunbowale, Elka R. Georgieva

**Affiliations:** Department of Chemistry and Biochemistry, Texas Tech University, Lubbock, TX 79409, USA; aogunbow@ttu.edu

**Keywords:** *E. coli* expression host of heterologous transmembrane proteins, transmembrane protein fusion strategies, protein engineering, soluble transmembrane proteins

## Abstract

To delve into the structure–function relationship of transmembrane proteins (TMPs), robust protocols are needed to produce them in a pure, stable, and functional state. Among all hosts that express heterologous TMPs, *E. coli* has the lowest cost and fastest turnover. However, many of the TMPs expressed in *E. coli* are misfolded. Several strategies have been developed to either direct the foreign TMPs to *E. coli*’s membrane or retain them in a cytosolic soluble form to overcome this deficiency. Here, we summarize protein engineering methods to produce chimera constructs of the desired TMPs fused to either a signal peptide or precursor maltose binding protein (pMBP) to direct the entire construct to the periplasm, therefore depositing the fused TMP in the plasma membrane. We further describe strategies to produce TMPs in soluble form by utilizing N-terminally fused MBP without a signal peptide. Depending on its N- or C-terminus location, a fusion to apolipoprotein AI can either direct the TMP to the membrane or shield the hydrophobic regions of the TMP, maintaining the soluble form. Strategies to produce G-protein-coupled receptors, TMPs of *Mycobacterium tuberculosis*, HIV-1 Vpu, and other TMPs are discussed. This knowledge could increase the scope of TMPs’ expression in *E. coli*.

## 1. Introduction

Membrane proteins fulfill vital physiological functions in all living organisms and, in some cases, are involved in causing disease conditions due to, for example, mutations [1,2,3,4]. Our focus here is on transmembrane proteins (TMPs), which constitute about 60% of pharmacological targets [5,6,7]. Because of their essential roles, acquiring detailed knowledge of these proteins’ molecular mechanisms is vital to gain control over their functions, e.g., designing inhibitors of TMPs encoded by pathogens or characterizing and possibly reversing protein malfunction. In this regard, in vitro studies are important, particularly obtaining the high-resolution structure of TMPs, studying their interactions with ligands, elucidating the conformational rearrangements taking place during their function, and conducting functional assays [8,9,10]. However, these in vitro studies typically require large quantities of highly pure TMPs. To obtain the highly pure TMPs, heterologous expression in a host organism, such as *E. coli*, yeast, insect, and mammalian cells, is typically required [11,12,13,14]. Among these, *E. coli* is the most extensively used protein expression host because of its relatively low cost, rapid expression rate, and easy genetic manipulations [15,16,17,18]. *E. coli* is particularly suitable for producing bacterial TMPs. However, some extraneous bacterial TMPs have been found in the insoluble inclusion bodies when expressed in *E. coli*, e.g., *Mycobacterium tuberculosis* (*Mtb*) TMPs [19]. Furthermore, when expressed in *E. coli*, eukaryotic and viral TMPs are almost inevitably aggregated since they require different than *E. coli*’s translocon and chaperone systems for delivery to the membrane and folding [20]. Refolding and purifying these aggregated proteins is laborious and often inefficient [17,21]. To overcome these obstacles in TMPs’ production, protein engineering has been advanced toward designing and expressing in *E. coli* chimeric protein constructs containing a specific protein tag fused to the target TMP. This allows directing the TMPs of interest to the *E. coli* plasma membrane or maintaining it in soluble form [20,22,23,24,25,26]. Such approaches provide new possibilities to study the structure and function of TMPs, which aid the understanding of physiological mechanisms and pharmacological developments.

This review focuses on some of the most successful TMPs’ fusion strategies, which made it possible to express eukaryotic, viral, and prokaryotic TMPs in the easy-to-handle state in *E. coli*. The development and applications of fusion tags such as signal peptide and signal peptide with maltose binding protein (MBP), mistic protein, apolipoprotein AI, and mature MBP (without signal peptide) are summarized in Table 1 and discussed in greater detail in the following sections of this review paper.

## 2. Fusion Strategies to Produce Heterologous Transmembrane Proteins in *E. coli*

### 2.1. Fusion Proteins Aid the Insertion and Folding of Heterologous TMPs in the E. coli Plasma Membrane

#### 2.1.1. Signal Peptides and Precursor Maltose Binding Protein Fusion Strategies

The fusion of a signal peptide to the N-terminus of eukaryotic TMPs was among the earliest strategies to produce these proteins in *E. coli*. Typically, upon synthesis, eukaryotic, viral, and some bacterial TMPs are not recognized for membrane insertion and end up in a misfolded inclusion body state [27,35]. However, the addition of short 20–30 amino acids signal peptides to the target protein’s N-terminus makes the protein recognizable by the *E. coli* machinery for trafficking to the plasma membrane [12]. Therefore, for expression in *E. coli*, the periplasmic leader sequences derived from ompT, ompA, pelB, phoA, malE, lamB, β-lactamase and PelB can generally be used to direct eukaryotic TMPs to *E. coli*’s plasma membrane [12,36]. In this case, the signal peptide–TMP polypeptides are translocated post-translationally via the Sec-dependent pathway. Conversely, the native to *E. coli* TMPs have highly hydrophobic signal peptides and are translocated via the SRP-dependent pathway utilizing a co-translational mechanism. These hydrophobic signal peptides (e.g., the peptide derived from the DsbA protein) can also be used as an N-terminal tag to express heterologous TMPs [36].

**Figure 1:**
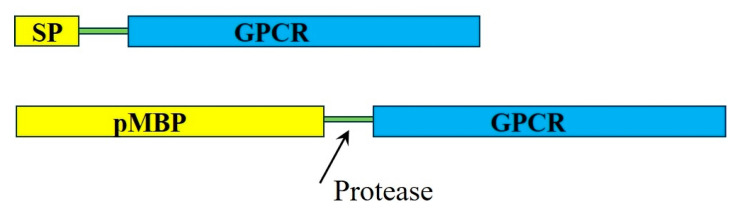
Schematic representation of the pMBP signal peptide (SP)-GPCR and pMBP-GPCR chimera constructs used to produce functional GPCRs in *E. coli* plasma membranes. In some cases, a protease site between pMBP and GPCR was introduced to remove the tag after purifying the protein.

The application of the malE (maltose binding protein, MBP) signal peptide has been successful in the production of several members of the G protein-coupled receptors (GPCRs) family. In these studies, the peptide containing the signal sequence for periplasmic localization of the *E. coli*-encoded MBP, or even the entire MBP with the signal peptide included (the precursor MBP, pMBP), was fused to the N-terminus of GPCRs (Figure 1). This chimeric construct was directed to the plasma membrane, where it adopted a natively folded and functional state [28,30,37]. Initially, this method was used to express in *E. coli* serotonin 5-HT1A and neurotensin receptors in a membrane-bound state [27,28]. Later, the strategy was applied to several other GPCRs, such as the rat NK-2 (neurokinin A) receptor [29], rat neurotensin receptor [37], M2 muscarinic acetylcholine receptor [30], peripheral cannabinoid receptor [31], and others. The success of these studies was partly due to the extracellular localization of the GPCRs’ N-terminus, which allowed the MBP signal sequence to direct this protein region to the *E. coli* periplasmic space and ensure proper orientation of the first TM helices of the receptors [28,38]. These advancements were instrumental in progressing GPCRs’ structural and functional studies, aiding pharmacological developments. In their original work, Henderson and colleagues and later studies [28,39] found that the expressed in *E. coli* membranes neurotensin receptor with an N-terminus fusion signal sequence with and without the entire MBP could bind the ligand neurotensin. However, the presence of pMBP significantly increased the receptor–ligand affinity. After that, the high-resolution structures of GPCRs produced in *E. coli* were solved, thus further enhancing the understanding of these proteins’ structure–function relationship. As a result, multiple X-ray structures of neurotensin receptor one was solved at high resolution [39]. Furthermore, the high-level functional GPCRs’ expression in *E. coli* have greatly facilitated NMR studies of these proteins as well, providing structural and dynamic insights underlying the interaction with agonist and antagonist molecules [40,41].

All these studies were based on a similar construct design and cloning in the *E. coli* expression vectors pRG/II-pMBP or pRG/III-hs-pMBP created in the original studies of neurotensin under the control of *lac* promoter and IPTG induction [28,29,37]. The original vector containing the Thrombin (Thr) cleavage site to remove the tag was further replaced by a more selective HRV 3C protease site because Thr was found to aggregate the GPCR [42]. In addition to protein engineering to incorporate a signal peptide, the high-yield production of functional GPCRs in *E. coli* was improved through the optimization of protein expression temperature (typically at 22 °C or lower) and concentration of IPTG (typically low concentration of 0.1–0.3 mM was used) [30,31].

#### 2.1.2. Mistic Protein Fusion Strategies

**Figure 2:**
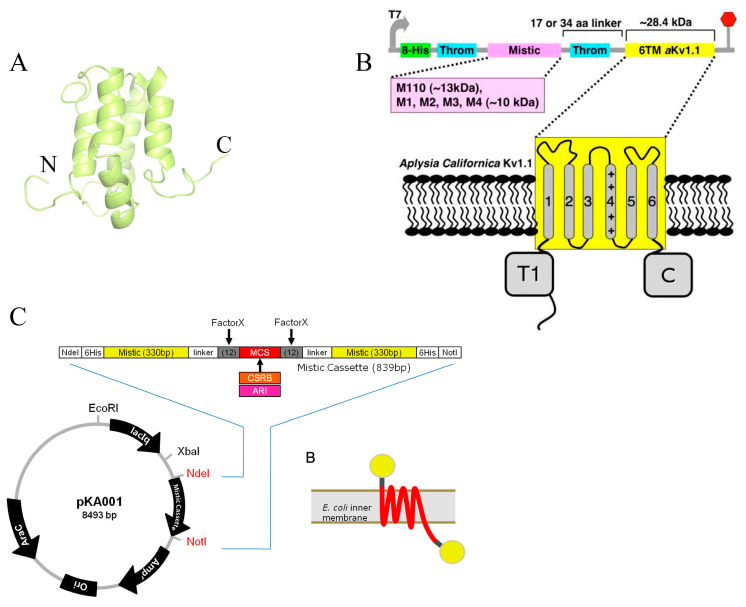
Mistic fusion strategy to produce heterologous TMPs in *E. coli*. (**A**) NMR structure of mistic, PDB code 1YGM. The N- and C-termini are indicated. The protein folds into a 4-helix bundle. (**B**) Fusion strategy used to produce the aKv1.1 channel and aKv1.1 6TM. The figure was adopted from Ref. [20] with permission from Elsevier (License number 5665041200130). Only one mistic copy was fused to the N-terminal of aKv1.1. (**C**) Fusion strategy to produce eukaryotic rhodopsin variants. The figure adopted from Ref. [22] with permission from Elsevier (license number 5665050773775). Two copies of mistic were fused to the N- and C-termini of the TMPs.

In other studies, the mistic protein fused to the N-termini of eukaryotic TMPs for expression in *E. coli* was utilized [20,43]. Mistic (an acronym for “membrane-integrating sequence for the translation of integral membrane protein constructs”) is encoded by *Bacillus* species and was originally found in *Bacillus subtilis* [44,45]. The protein folds into a four-helix bundle with a hydrophobic core and a significant fraction of polar and charged amino acids (Figure 2A) [44]. Mistic is found in both cytoplasmic and membrane-bound states [20,46]. The mistic protein of *Bacillus subtilis* (M110) comprises 110 amino acid residues and has a net charge of −12.0 at pH 7. It has been suggested that its acidic nature enables the tight association with the lipid bilayer alone or as a fusion tag when expressed in *E. coli* [20]. However, the shorter than M110 mistic constructs or orthologs found in other species with also highly acidic nature, e.g., the 84 amino acids C-terminal truncated version of M110 (referred to as M1) as well as mistic from *B. leicheniformis* (referred to as M2) and from *B. mojavensis* (referred to as M3), are highly soluble with almost exclusive cytoplasmic localization.

In contrast, the mistic from *B. atrophaeus* (M4) is comparable to M110 membrane affinity [20]. Interestingly, outside the membrane, soluble mistic forms fibrils with a protomer’s structure that is largely different from those determined by NMR for non-fibrinous mistic [44,45]. The fibrous structures possibly shield the charged regions of mistic and facilitate its interaction with hydrophobic membranes [45]. To determine the membrane association regions, Marino et al. analyzed truncated mistic constructs containing individual or combined helices. They found that helices 1, 2, and 4 interact with lipid membranes, whereas helix 3 is primarily soluble [46]. It was found in the same study that the single helices 1, 2, and 4 fused to the N-terminus of Y4 GPCR can direct the protein to the *E. coli* membrane [46], similarly to full-length (FL) mistic [47]. However, only the fusion of Y4 GPCR to helix 2 yielded an expression level comparable to those when FL mistic was used and a segment of amino acids “GLDAFIQLY” in helix 2 was identified as the minimal sequence for mistic and its fusion protein to interact with the membrane [46].

It has been proposed that the absence of a detectable signal sequence, which is a unique feature of mistic, enables this protein to avoid the Sec translocon’s pathway of *E. coli*; due to this, mistic’s and mistic-tagged TMPs’ expression does not overload the protein translation machinery [20,44]. Therefore, high expression yields of heterologous TMPs in mistic-tagged TMP chimeras can be achieved [20,48]. It has also been reported that mistic facilitates the expression of functional proteins with both the N-terminus inside or N-terminus outside the cell [43,49], suggesting its adaptive membrane-bound topology to accommodate the expression and folding of the target protein.

In addition to GPCR [46,47], the aKv1.1 channel and its six-transmembrane helix (6TM) domain have also been successfully produced in *E. coli* as mistic–fusion constructs (Figure 2B) [20]. It was found that the expression of the aKv1.1 6TM and shortening of the mistic–aKv1.1 6TM linker had a positive effect on the target protein expression levels due to possibly better interaction between mistic’s C-terminus and aKv1.1 6TM as well as reduced proteolysis in the linker region [20]. It was further established that the fusion of aKv1.1 6TM to the C-termini of mistic M110 and mistic M4 resulted in comparable expression levels, which was possibly because both M110 and M4 aided the membrane insertion of aKv1.1 6TM similarly [20].

The mistic fusion strategy has facilitated the studies of the eukaryotic type I rhodopsin as well because it enabled the economical production of the functional form of this protein in *E. coli*. [22]. Interestingly, this study found that two mistics copies fused to the N- and C-termini of the target proteins were needed to direct them into the *E. coli* membranes; the study was conducted on several eukaryotic rhodopsin variants, including ARI and CSRB, as well as other eukaryotic TMPs (Figure 2C) [22]. It was also found in this study that the mistic moieties of the fusion construct do not severely affect the proton transport function of the ARI [22], which might be advantageous as the expression level of some heterologous TMPs is relatively low, and the removal of fusion tag typically leads to a further reduction in protein quantities.

Expressed in *E. coli* and purified mistic-tagged eukaryotic proteins were also used as antigens for raising polyclonal antibodies, and it was found that the mistic–TMPs antibodies recognized the corresponding TMPs in native membranes more efficiently than the antibodies raised against just the soluble domains of these TMPs [48]. As the study’s authors suggest, this could be because the soluble domains of the studied TMPs might adopt a distinct conformation when included in the membrane-bound FL protein vs. truncated soluble versions [48].

Generally, the use of mistic for the expression of different membrane proteins depends on the target protein’s proteolytic susceptibility, the protein expression induction conditions, and the number of amino acids that connect the mistic to the recombinant membrane protein [44]. Mistic’s structure and membrane affinity are critical for their ability to facilitate the production of heterologous TMPs [44]. This was confirmed by the work of Tarmo and colleagues, who used three mutant variants of mistic protein (W13A, Q36E, and M75A) with amino acid substitutions in different helical regions. The expression level of the mutants in the cytoplasm and membrane were tested when alone and fused to aKv1.1. It was seen that the mutation of methionine-75 to alanine destabilized the structure of mistic protein due to its substantial partitioning between the membrane and cytoplasm. Also, when the mutant was fused to aKv1.1, there was no expression of this protein in the membrane [44].

The mistic protein can also be combined with another fusion protein to increase the expression rate of some TMPs. Ananda et al. discovered that the *CB2* gene can be expressed only when mistic and TarCf are fused to its N- and C-terminus, respectively, indicating a synergistic effect of the two tags on the expression [43].

#### 2.1.3. Apolipoprotein A-I Fusion Strategy

Apolipoprotein A-I (apoAI) belongs to the spherical high-density lipoproteins (HDLs), which are abundant in human plasma. ApoAI is a highly α-helical protein of 28 kDa, which in vivo serves as a “glue” to hold HDL particles together [50]. The protein is easily produced in *E. coli*. It has been widely utilized in structural and functional studies of membrane-reconstituted TMPs as the tertiary complex of target TMP-lipid-apoAI form discoidal nanoparticles stabilized by a double belt of apoAI [8,51,52,53].

Recently, motivated by a study on soluble TMPs fused to the N-terminus of apoAI (discussed in more detail below) [26], our research group designed and expressed in *E. coli* a chimera construct of apoAI with the *Mycobacterium tuberculosis* EfpA (*Mtb*-EfpA) drug exporter [32]. By doing so, we produced, for the first time to the best of our knowledge, highly pure FL *Mtb*-EfpA in quantities sufficient for downstream in vitro characterization. Remarkably, when reconstituted in lipid, because of the presence of apoAI in the apoAI–EfpA fusion construct, we observed by electron microscopy the formation of protein–lipid nanoparticles [32], which are similar to previously described nanodiscs [51,52,53]. This suggests that we can carry out future studies on EfpA’s properties (e.g., drug binding, structure determination, assessing the conformational dynamics) using these two-component (apoAI-EfpA protein and lipid) nanoparticles. Moreover, the methodology could also be adopted in studies on other TMPs.

Interestingly, apoAI is typically expressed as a soluble protein in *E. coli* [54]. We also found that the untagged EfpA is deposited in inclusion bodies upon expression [32]. Therefore, there is a question of how and why the apoAI–EfpA is directed to the membrane. One explanation could be that the additional sequence at EfpA’s N-terminus prevents the protein from misfolding at the stage of protein translation as was previously proposed for mistic’s mechanism to prevent TMPs’ aggregation [45]. Similar effects on protein expression were also observed when TMPs were tagged at their N-termini with glutathione S-transferase (GST) [55] or YbeL and YnaI [56].

### 2.2. Fusion Proteins Aid the Production of Heterologous TMPs in Soluble Form in E. coli

In addition to membrane-bound heterologous TMPs, some TMPs or their transmembrane portions have been produced in *E. coli* in soluble form. Some of these developments are discussed below.

#### 2.2.1. Mature (without Signal Peptide) Maltose-Binding Protein Fusion Strategies

The mature MBP (mMBP) lacking the signal peptide to direct it to the periplasm has also been used to produce a range of TMPs in *E. coli*, which remain inside the cell due to the absence of the MBP signal sequence. These TMPs were commonly small and obtained in soluble form, but some proteins or protein fractions were also found in the inclusion bodies [19,24,33,34,57,58]. In addition to serving as a solubilization tag, MBP is also very useful as a purification affinity tag, which, in combination with a polyhistidine tag for Ni-affinity purification, makes it a powerful tool to isolate proteins of high purity [24,59].

Some studies suggest that the solubility-enhancing property of mMBP is mediated by its open conformation of the ligand-binding cleft state [23,60,61,62]. However, the exact mechanism by which mMBP increases the solubility of recombinant proteins needs to be better understood. Lebendiker and colleagues suggested that mMBP can act either as a magnet that attracts chaperones toward the environment of the recombinant protein or by forming micelle-like aggregates that hold incompletely folded proteins [61]. It was also proposed that mMBP acts as an electrostatic shield by reducing the electrostatic repulsion between highly charged soluble polypeptide extensions, thereby reducing the chance of repulsion [61,63]. One plausible explanation for the increased solubility of the mMBP-tagged TMPs is that the located at the N-terminus MBP moiety is translated first from the ribosome and becomes fully folded before the TMP is translated [24,64], but to whether the TMP is natively folded or just held by mMBP in the solution could depend on the particular protein and needs further characterization.

mMBP fusion was instrumental in producing several TMPs of *Mtb* for structural studies by NMR [19,57]. As discussed above, when expressed in *E. coli*, *Mtb*’s TMPs are typically deposited in the insoluble fraction. Therefore, using a mMBP solubilization tag fused to the N-termini of the TMPs of interest proved helpful in producing these proteins. It is tempting to mention that mMBP-tagged TMPs of *Mtb* were not found in the *E. coli* plasma membrane [19,57], which is the opposite to what we observed in the case of apoAI–EfpA [32]. These differences might be because of the TMPs size, i.e., single-pass small TMPs [65] vs. large multi-pass TMP [66], but future examinations might be needed to understand this better.

A study of a truncated form (p18) of the Bax apoptotic protein found that the highly hydrophobic and membrane-residing p18 can be expressed and handled in soluble form when fused to the C-terminus of mMBP [33]. This soluble form was competent in interacting with the membrane of isolated liver mitochondria and triggering cytochrome c release in a dependent manner [33].

An interesting study was reported about catalytically active soluble oligomers of mMBP-tagged YqgP protease, which could cleave a substrate within the transmembrane domain [34]. YqgP is a membrane-residing rhomboid protease homolog believed to have an active site in the membrane interior [67,68]. It may well be that the YqgP’s oligomerization outside the membrane protects the hydrophobic protein regions from the aqueous environment and provides conditions similar to the membrane for assembling the enzymatic site. Although no structural information about these soluble oligomers was delivered, one would expect that the protein monomers should have similar conformations in soluble oligomers and membranes to maintain the activity.

**Figure 3:**
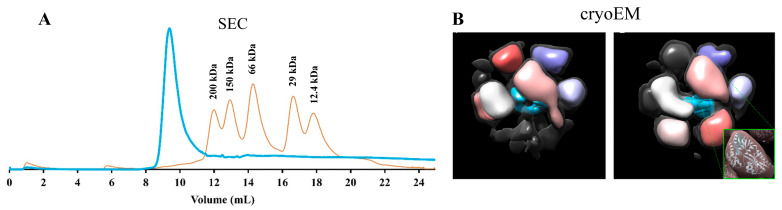
MBP-Vpu chimera construct forms soluble oligomers. (**A**) Size exclusion chromatography (SEC) of purified 50 µM MBP-VPu (blue) shows that the protein forms oligomers with molecular weight greater than 250 kDa. The SEC of a mixture of protein molecular weight standards is in white brown, and the peaks corresponding to proteins with different molecular weights are indicated. The figure was adopted from Ref. [24] with permission from Elsevier (license number 5665051248242; accessed on 9 November 2023). (**B**) cryoEM analysis of soluble MBP-Vpu oligomers revealed predominantly hexamers and hexamer-to-heptamer equilibrium. The MBP moieties of each MBP-Vpu monomer are colored in pink, gray, shades of purple, and red. The electron density, which most likely represents the Vpu oligomerization core, is colored in blue. The figure was adopted from Ref. [25] under the conditions of the Creative Commons Attribution 4.0 International License (https://creativecommons.org/licenses/by/4.0/; accessed on 9 November 2023).

Similarly to YqgP protease, our lab recently found that the HIV-1 Vpu protein also forms soluble oligomers when expressed as a fusion construct with mMBP (Figure 3) [24,25]. Previously, Vpu was exclusively considered an TMP. Although the possible physiological role of these soluble Vpu oligomers is currently unknown, the existence of such a role cannot be ruled out. Significantly, the soluble Vpu could interact with membranes undergoing conformational changes.

All these examples show that the fusion strategy with mMBP successfully aids the production in *E. coli*, where it is otherwise difficult to obtain heterologous TMPs to facilitate downstream investigations on these proteins. The discussed studies also demonstrated that the TMPs produced in soluble form retain activities in terms of being able to interact with membranes and fulfill their catalytic functions [24,33,34]. Therefore, mMBP can be used as a powerful protein engineering tool to manipulate the TMPs’ production in *E. coli*, but it could be utilized in enzyme immobilization for biotechnological applications [69].

#### 2.2.2. Apolipoprotein A-I Strategies to Produce Soluble TMPs

**Figure 4:**
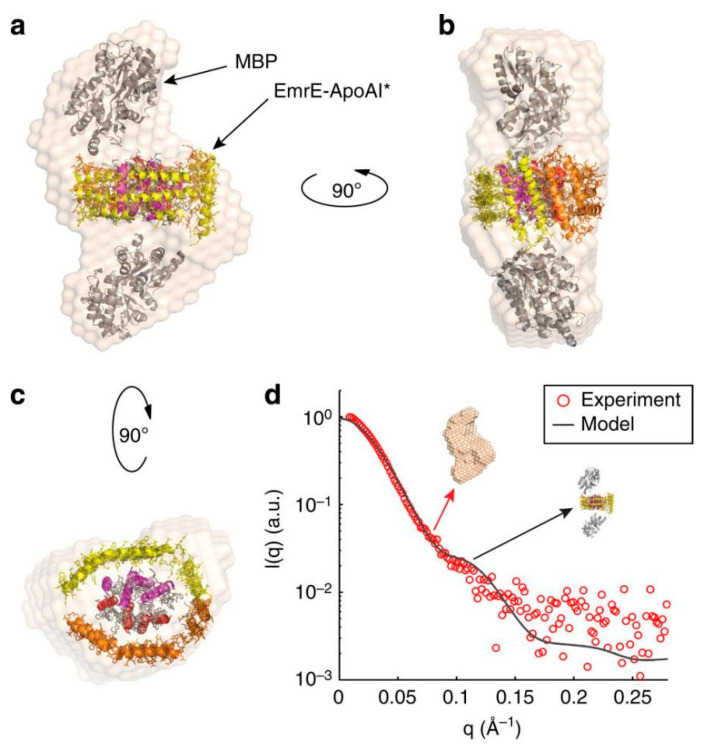
Soluble apoAI–EmrE studies by SAXS. (**a**–**c**) Multiple views of the reconstructed particle envelope calculated ab initio from the dimer SAXS data (red circles in (**d**)) using DAMMIF [70]. The asterisk (*) in (**a**) denotes ApoAI lacking its 43-residue globular N-terminal domain. (**d**) Comparison between the experimental scattering profile of the dimer (red circles) and the theoretical profile calculated for the proposed model using CRYSOL software version 3.0 (solid line). The figure was reproduced from Ref. [26] under the conditions of Creative Commons Attribution 4.0 International License. Note, in addition to the C-terminal apoAI tag, the EmrE protein had an N-terminally fused mMBP tag as well. The dimeric state of EmrE was preserved in the soluble form.

A study by Mizrachi and colleagues has described a method to obtain soluble TMPs by fusing them to the N-terminus of truncated apoAI [26]. The authors found that apoAI forms a scaffold around the hydrophobic TMP’s regions to shield them from the water environment and stabilize the TMP-apoAI complex. Multiple prokaryotic and eukaryotic TMPs, including single and multi-pass and oligomeric TMPs with C-terminal apoAI tags, were produced in substantial quantities in *E. coli* in soluble form outside the membrane. Furthermore, these apoAI-scaffolded TMPs were stable and uniform, and they were characterized by small angle X-ray scattering (Figure 4). Remarkably, the authors found that the solubilized EmrE transporter retains ligand-binding activity. Due to the fact that a large number of TMPs were studied, this methodology promises that many other TMPs can be expressed in *E. coli* in soluble form, and further functional (e.g., ligand binding and protein–protein interaction) and structural studies can be conducted on them. It is worth mentioning that these apoAI-tagged proteins were not directed to the membrane, which was possibly because the apoAI was expressed after the corresponding TMP. Also, care was taken that the prokaryotic TMPs were genetically modified and/or mMBP was added to their N-termini to keep them in the cytoplasm [26].

#### 2.2.3. Other Protein Design Strategies to Produce and Stabilize Soluble TMPs

In addition to fusion tags, other protein engineering strategies have helped to overcome the challenges imposed by the low expression and instability of TMPs produced in *E. coli*. In the last decades, multiple approaches with different levels of applicability have been reported. Here, we highlight just two examples.

Protocols for enhancing the expression level of TMPs in *E. coli* and improving the stability of the purified protein for structure determinations by termini restraining tags (such as variants of green fluorescence protein, thioredoxin or T4 lysozyme) were also developed [71]. In this recent study, along with TMPs expressed in *Pichia pastoris* and mammalian cells, prokaryotic proteins, such as DsbB cloned in the pET28b vector, were expressed in *E. coli*, and their structure successfully was determined [71].

“Solubilization by design” was used to produce in *E. coli* soluble dimers of motility protein B (MotB) [72], which is a component of bacterial flagellum [73]. In this study, the two transmembrane helices of the MotB dimer were replaced by a leucine zipper; the dimers were stable and monodisperse, and they were composed of adequately folded subunits; these engineered MotB dimers were of high quality for crystallization and structure determination [72].

A 24-amino acids peptide was designed to form an amphipathic helix with a “flat” hydrophobic surface that would interact with a transmembrane protein as a detergent [74]. Alone, the peptide forms a homo-oligomeric 4-helix bundle with a helix length of 30 Å, which is sufficient to traverse the membrane; when the peptide was mixed with bacteriorhodopsin and rhodopsin, a large percentage of these TMPs (>60%) remained in solution even without detergent [74].

## 3. Conclusions

Unlike soluble proteins, the expression, purification, and characterization of TMPs is notoriously difficult and expensive. To minimize the cost and time for heterologous TMPs’ production, *E. coli* has become a host of choice for producing these proteins. However, due to physiological differences in translocon systems among TMPs’s original organisms and *E. coli*, many of the proteins of interest end up in a misfolded and challenging-to-handle state because *E. coli*’s TMP membrane-targeting machinery does not recognize them. Furthermore, the refolded from this aggregated state TMPs often have low or no activity. Several strategies to produce heterologous TMPs in *E. coli* in either membrane-bound or soluble form have been developed to tackle these problems. Most of these strategies require thoughtful protein engineering to select a fusion tag with particular properties and link this tag to either the N- or C-terminus of the TMP of interest.

This review summarized the progress made in producing several eukaryotic, viral, and prokaryotic TMPs tagged with either MBP, apoAI, or other proteins. Along with providing rationales for how the fusion tag can affect the expression of TMPs, we described several examples of produced and studied TMPs. These successful scientific stories provide unambiguous evidence that the developed methodologies have been instrumental in studies of key physiologically and pharmacologically important TMPs. The progress made so far lays a solid foundation for further advancements in membrane biology to explore the fascinating mechanisms of TMPs from diverse organisms.

## Figures and Tables

**Table 1:** Fusion tags, the transmembrane proteins (TMPs) produced using them, the benefit of these fusion strategies for TMPs structural and/or functional studies, and the reported TMPs’ yields are listed.

Fusion Tag	Produced TMP	Benefit for Structural and/or Functional Studies	Protein Yield
MBP signal peptide/entire MBP	Serotonin 5-HT1A [27], neurotensin receptor [28], NK-2 (neurokinin A) [29], M2 muscarinic acetyl choline receptor [30], and peripheral cannabinoid receptor [31]	It promotes the proper folding and insertion of the recombinant fusion protein into the plasma membrane. It supports the application of functional assays in the study of the activities of the transmembrane protein.	Yeliseev et al. (2005) showed that the small scale purification yielded 1–2 mg of recombinant receptor/1 L of culture [31]
Mistic protein	aKv1.1 channel [20], and eukaryotic type I rhodopsin [22]	It promotes high expression yield of heterologous TMPs as well as facilitating the expression of functional proteins with both N-terminus inside or N-terminus outside.	The yield of Mistic-aKv1.1 was approximately 2 mg/L culture [20] The Mistic-ARI yield amounted to roughly 0.12 mg/L culture [22]
Apolipoprotein AI	Mtb-EfpA [32], EmrE transporter [26], human cyt b5 [26], HSD17β3 [26], GluA2 [26], DsbB [26], CLDN1 [26], CLDN3 [26], S5ɑR1 [26], S5ɑR2 [26], NRC-1bR [26], OmpX [26], and VDAC1 [26]	The tertiary conformation of the TMP–lipid–apoAI forms a discoidal nanoparticle stabilized by a double belt of apoAI It increases the solubilization of TMPs with high levels of expression and supports the functional study of the protein (e.g., ligand binding and protein–protein interaction).	Mizrachi et al. (2015) showed that the diverse range of IMPs yielded approximately 5–10 mg/1 L of culture [26]
mMBP without signal peptide	Vpu [24], p18 [33], and Yqgp protease [34]	It is useful as a purification affinity tag when in combination with polyhistidine tag for Ni-affinity purification. It is a natural fusion tag that is a solubility enhancer.	The approximate yield of MBP-p18 reached around 20mg/L culture [33] The yield of MBP-Yqgp protease was 2 mg/1 L of culture [34]

## Data Availability

Data sharing is not applicable to this article as no new data were created or analyzed in this study.
